# Editorial for the Special Issue on 3D Printed Actuators

**DOI:** 10.3390/mi14010077

**Published:** 2022-12-28

**Authors:** Ed Habtour, Samuel Stanton

**Affiliations:** 1 William E. Boeing Department of Aeronautics & Astronautics, University of Washington, Seattle, WA 98195-2400, USA; 2 Aeronautics Department, United States Air Force Academy, Colorado Springs, CO 80840-5002, USA

After decades of curiosity-driven innovation and engineering advancements of 3D-printed actuators, we continue to witness their endless impacts and ever-expanding opportunities in many applications that touch our lives. It is fair to say that the 3D printer has been monumental in bringing together seemingly disparate disciplines to solve various challenges; undoubtedly, 3D-printed actuators have extended the frontiers of knowledge in areas such as engineering, chemistry, biology, physics, mathematics, and arts. One particular challenge is the creation of actuators rivaling the dynamic performance, structural stability, and energy efficiency in animals. The research findings reported in the 3D-Printed Actuators Special Issue show promising approaches for engineering biology-like actuation. These findings have led to the following: the creation of an anatomically correct robotic hand with human-like grasping capabilities [1]; emulation of an antagonistic variable stiffness actuation observed in the musculoskeletal system of snakes [2]; reducing friction in rotating parts and joints by combining 3D printing and radiation pressure [3]; fabrication of flexible piezoelectric composite actuators without application of any adhesives [4]; extension of the service life of actuators through geometric and material optimization [5]; and acceleration of print inspection through an open-source low-power computer-vision hardware [6].

The promising capabilities of 3D-printed actuators reported in this issue, such as structural flexibility, high energy efficiency, and environmental adaptability, will facilitate incredible possibilities for actuating wearables, haptic devices, prosthetics, and biomimetic robots. In the field of anthropomorphic (human-like) robotic design, Tian et al. proposed a 3D hand model to enable a single material 3D printing in one go of a robotic hand with the full degrees of freedom (DoFs) of a human hand, and artificial bones and ligaments [1]. Wiersinga et al. printed a musculoskeletal system with hybrid stiffness compliance to emulate the antagonistic interactions between the elastically rigid segments (bones) and soft tissues (muscles and tendons) required for high DoFs and efficient undulations in snakes [2]. The rigid–soft interactions provide a remarkable increase in the energy efficiency by nearly 68%, compared to 2.5–30% efficiency in purely soft actuators.

While increasing DoFs through segmentation increases the range of motion, it poses other challenges, including the presence of frictional forces between the joints, and the need for adhesives to bone incompatible elastic and soft materials. Arai et al. successfully reduced frictions generated by rotation parts by using optical radiation pressure during the printing process. The circumferential gaps in and between the printed parts were exposed to light [3]. The radiation pressure generated by multiple reflections of the light within the gaps smoothed the circumferential surfaces. Current techniques for fabricating flexible piezoelectric (PZT) actuators involve using adhesives to bond PZT to elastic or soft materials. The typical fabrication involves complex manufacturing processes such as photolithography, etching, sol-gel, and sputtering. Using a cost-effective electrohydrodynamic (E-Jet) printing, Wang et al. applied PZT film directly to elastic structures, followed by co-firing sintering to form PZT actuators [4]. Their method eliminated the use of adhesives—thus overcoming the limits of voltage drops, and absorbing amplitude in current PZT actuators.

The endurance of 3D-printed actuators, especially soft actuators, is a well-known engineering challenge. Optimizing their endurance was demonstrated by Fateri et al. In their study, they combined the material stiffness and geometric design optimization to extend the lifetime of actuators. Their approach was demonstrated experimentally using 3D-printed pneumatic actuators. We are also pleased to include Gervasi et al.’s design of a stand-alone open-hardware microscope device, which can automatically collect samples with 3D-printed pumps, and can capture images at up to 50× optical magnification with a digital camera. The images can be stored and analyzed using computer vision algorithms, which require a low-power integrated single-board computer. The modular design can be freely reproduced with user-friendly components such as Arduino, Raspberry pi, and 3D printers.

Finally, it is important to emphasize that our special issue not only highlights the many incredible possibilities in the field of 3D-printed actuators, but also challenges our current engineering thinking. The convergence of exciting advances in 3D printing, combined with the intersecting frontiers of various scientific disciplines, is enabling us to discover and exploit new nonlinear behaviors required for realizing dynamics and functions rivaling biology. Creating nature-like actuation systems whose structures are composed of soft–rigid interfaces is no longer a mere possibility, but instead a reality.
